# Serum growth differentiation factor-15 (GDF-15) is a biomarker of cardiac manifestations in children with COVID-19

**DOI:** 10.1186/s40001-023-01514-8

**Published:** 2023-11-16

**Authors:** Sally Raafat Ishak, Mona Mostafa El Ganzoury, Eman Mahmoud Fouda, Maha Ahmad Anwar, Amany Moustafa Kamal, Heba Mostafa Hamza, Nehad Ahmed Bakry

**Affiliations:** 1 Pediatrics Department, Faculty of Medicine, Ain Shams University, Cairo, Egypt; 2 Clinical Pathology Department, Faculty of Medicine, Ain Shams University, Cairo, Egypt; 3 Faculty of Medicine, Menoufia University, Menoufia, Egypt

**Keywords:** COVID-19, Heart, GDF-15, Biomarkers, Child

## Abstract

**Background:**

COVID-19 leads to severe overwhelming inflammation in some patients mediated by various cytokines (cytokine storm) that usually leads to severe illness accompanied by cardiovascular manifestations. Growth differentiation factor-15 is a cytokine induced by stress and is associated with inflammatory processes in the lung and heart. This study aimed to measure the level of serum growth differentiation factor (GDF-15) in children with COVID-19 and to correlate it with the disease severity, cardiac affection, and the outcome of COVID-19.

**Methods:**

A cross-sectional study was conducted on 144 children; 72 children diagnosed with COVID-19, and 72 healthy children. The severity of COVID-19 was assessed clinically, laboratory, and radiologically. Echocardiography was done within 48 h of admission for COVID-19 patients. Serum GDF-15 was measured by ELISA for both patients and controls.

**Results:**

Serum GDF-15 level was significantly higher in patients with COVID-19 than in controls (p < 0.01). In COVID-19 patients with severe clinical grading, those who were hospitalized in the PICU, and those who died, serum GDF-15 levels were greater. individuals with cardiac manifestations exhibited significantly higher serum GDF-15 levels than individuals without them. In children with COVID-19, increased GDF-15 was correlated to poorer ejection fraction and higher INR using multivariate linear regression analysis.

**Conclusion:**

Serum GDF-15 is a promising biomarker of COVID-19, it can be used as a predictor of cardiac manifestations in children with COVID-19 and severe disease.

## Introduction

COVID-19 infection can cause detrimental effects on the cardiovascular system and result in complications that include myocardial injury, arrhythmia, arterial and venous thromboembolism, myocarditis, cardiomyopathy, cardiogenic shock, and cardiac arrest [1]. As a result, grouping patients into risk categories for suitable early anticoagulant or fibrinolytic therapy is difficult [2].

GDF-15 is a cytokine produced due to inflammation, tissue damage, and mitochondrial stress [29]. It belongs to the Transforming Growth Factor β (TGFβ) family of proteins [3]. In normal physiological conditions, GDF-15 mRNA is expressed by a wide variety of cells and tissues [4]. Furthermore, GDF-15 is increased in many different inflammatory diseases and infections. Its high plasma level during the cytokine storm of COVID-19 greatly increases the incidence of cardiovascular injury and can thus be considered as a general biomarker of disease severity, which is increasing with advanced disease and associated with all-cause mortality [5].

As a circulating myokine, GDF-15 is increased as a metabolic response to muscle-specific mitochondrial stress and muscle dysfunction in humans [6]. GDF-15 is expressed weakly in heart muscle under normal physiology; however, the production of GDF-15 is upregulated in cardiomyocytes following ischemia–reperfusion injury. The mechanical stress and injury on the myocardium induce the secretion and release of GDF-15 [3]. Also, The basal expression of GDF-15 is very low in skeletal muscles compared to other organs, but GDF-15 expression and secretion can be induced by various stress conditions. GDF-15 is thus considered a myokine and cardiokine [7]. It is also known to play a role in regulating appetite and energy balance, making it a key factor in metabolic and immune responses [8–10].

Recent research has shown that GDF15 levels increase in response to COVID-19 infection, particularly in adults [11], but its role in children is still not known. This study aimed to assess the levels of GDF-15 in children with COVID-19 as a biomarker of myocardial and muscular affection in those patients.

## Materials and methods

From December 2021 to March 2022, this observational cross-sectional study was conducted on 72 COVID-19 children and 72 healthy children (age and sex-matched with the study group) as a control group, at Children’s Hospital, Ain Shams University, Cairo, Egypt.

*Inclusion Criteria; Patient*: Children aged from 1 month to 18 years with confirmed COVID-19 infection using real time-PCR (RT-PCR) with clinical presentation and radiological findings of COVID-19 [11]. Convenience samples were collected from the hospitalized children in the isolation department. ***Controls:*** Pediatric children with no clinical or laboratory evidence of COVID-19 infection (negative RT-PCR). No history of contact with positive patients. No clinical evidence of a recent infection. Age and sex matched with the patients.

*Exclusion Criteria* Patients with inflammatory or rheumatic disorders or a previous history of cardiovascular diseases or thromboembolism.

Patients were subjected to history taking for personal data (age and sex), contact with a COVID-positive patient, duration of hospital admission, disease comorbidities, and symptoms at the onset of the disease. Patients were stratified according to severity into mild, moderate, severe, and critically ill [12]. Clinical examination was done for vital data and weight (plotted on weight for age Z-scores [13]. Investigations were done including complete blood count (CBC), C-reactive protein (CRP), liver and kidney functions, D-dimer, ferritin, creatine kinase MB (CK-MB), troponin I, prothrombin time (PT), partial thromboplastin time (PTT), and the international normalized ratio (INR). Computerized tomography (CT) chest was graded according to the COVID-19 Reporting and Data System (CO-RADS) score from 1–6 [14] and echocardiography (ECHO) was done for all COVID-19 patients.

### Assessment of serum level of GDF-15 by (ELISA) for both patients and controls

Serum level of GDF-15 for both patients and controls was done using a Human GDF-15 ELISA Kit, a sandwich ELISA assay, (Catalog No: E-EL-H0080, Elabscience Biotechnology Co., Ltd, Guangdong, China). The detection range of the kit is 23.438-1500pg/mL.

All reagents and samples were brought to room temperature before use. Then 100μL standard or sample was added to each well and incubated for 90 min at 37 ℃ then the liquid was removed from the wells without washing.

After that, 100μLBiotinylated Detection Ab was added to the wells and incubated for 1 h at 37℃ after covering the plate with the plate sealer. After the incubation time, the fluid was aspirated and the wash was done 3 times using the wash buffer.

After the wash step, 100μL HRP Conjugate was added to the wells. The plates were covered by the plate sealer and incubated for 30 min at 37 ℃. Then the fluid in the wells was aspirated and the wash was done 5 times using the wash buffer.

Then 90μL Substrate Reagent was added to the wells. The plates were covered with the plate sealer again and incubated for 15 min at 37 ℃ away from the light. 50μL Stop Solution was added. The optical density (OD value) of each well was determined at once, using a microplate reader set to 450 nm. The micro-plate reader was opened, preheated, and set to the testing parameter in advance.

To calculate the concentration of the samples, a standard curve was constructed by plotting the average OD for each standard on the vertical (Y) axis against the concentration on the horizontal (X) axis, and a best-fit curve was drawn through the points on the graph. The OD of each sample was blotted on the standard curve. The sample concentration was found according to the sample OD value by the Sample curve. If the concentration of a sample was above the detection range of the kit, the sample was diluted and retested and its concentration was multiplied by the dilution factor.

The levels of GDF-15 were compared between COVID-19 children and controls, then correlated with parameters of severity of COVID-19 clinically, laboratory, and radiologically.

### Sample size

Using the PASS11 (Powe Analysis and Sample Size) program for sample size calculation, setting power at 80%, alpha error at 5%, and reviewing results from previous studies that showed GDF-15 as a biomarker associated with pulmonary impairment in COVID-19 and so can potentially be useful in stratifying the severity of COVID-19 cases (ACUC = 0.72) [16]. Based on this finding a sample size of at least 50 cases was needed.

### Statistical analysis

Data were tabulated, and statistical analysis was performed using Microsoft^®^ Excel^®^ version 2010 and SPSS^®^ for Windows^®^ version 27.0 for data processing. Data were described as:

range, mean, and standard deviation (SD) (for numeric parametric variables); range, median, and interquartile range (IQR) (for numeric non-parametric variables); or percentage (for categorical variables). The student “t” test was used for the comparison of means of two independent groups (for parametric data), Mann Whitney test was used to calculate the difference between quantitative variables in not normally distributed data in two groups (for non-parametric data), Chi-square test (X2) was used to find the association between the row and column variables and comparison between qualitative variables of the studied groups. Correlation testing between variables was done by Pearson's correlation in the case of parametric quantitative data or Spearman’s in the case of non-parametric quantitative and qualitative data. A P-value of > 0.05 indicates non-significant results. A P-value of < 0.05 indicates significant results.

## Results

There was no statistically significant difference between patients and controls regarding age and sex, but there was a statistically significant higher GDF-15 level in patients with COVID-19 than in controls (p < 0.01) (Table 1).

**Table 1:** Statistical comparison between patients and control group regarding age, gender, and serum GDF-15 level

		Patients group No. = 72	Control group No. = 72	Test value	P-value	Sig
Age (year)	Median(IQR)	6.0 (2.38–10.63)	7.25 (5–10)	− 1.828‡	0.068	NS
	Range	0.17–16	3.25–14			
Sex	Males	37 (51.4%)	40 (55.6%)	0.251*	0.616	NS
	Females	35 (48.6%)	32 (44.4%)			
Serum GDF-15 level (pg/mL)	Median(IQR)	614.23 (338.67–1109.33)	144.66 (89.53–205.8)	−8.501‡	< 0.001	HS
	Range	47.47–2940.2	59.36–272.8			

*S* significant, *NS* non significant, *IQR* Inter quartile range. *GDF-15* Growth differentiation factor 15, *IQR* Interquartile range

^*^Chi-square test

^‡^Mann–Whitney test

P-value < 0.01: highly significant (HS), ˃0.05: non-significant (NS)

Children who had fatigue and dyspnea had significantly higher serum GDF-15 levels (P = 0.031, 0.043, respectively) than those without fatigue and dyspnea. Additionally, its level was significantly greater in underweight children than in children with normal weight (P = 0.043) and in children whose hospital stays were longer than 10 days (P = 0.023) (Table 2). The COVID-19 patients who were admitted to the PICU, with severe clinical grading and those who died had significantly higher GDF-15 than those admitted to the ward, having mild to moderate grading, and those who survived (Table 3).

**Table 2:** Statistical comparison according to symptoms and demographic data of studied children with COVID-19 as regards serum GDF-15 level

		Serum GDF-15 level		Test value‡	P-value	Sig.
		Median (IQR)	Range			
Fever	No	493.3 (235.92–2150.4)	235.92–2150.4	− 0.183	0.855	NS
	Yes	623.7 (342.06–1079.96)	47.47–2940.2			
Cough	No	588.59 (334.36–1373.58)	47.47–2940.2	− 0.423	0.673	NS
	Yes	668.89 (445.38–1038.84)	72.17–2402.6			
Dyspnea	No	531.88 (223.72–854.1)	47.47–2940.2	− 2.019	0.043	S
	Yes	829.64 (511.76–1390.82)	63.74–2402.6			
Loss of taste and smell	No	623.7 (342.06–1138.7)	47.47–2940.2	− 0.305	0.760	NS
	Yes	553.48 (335.28–1017.04)	187.52–2402.6			
Sore throat	No	581.39 (229.82–1444.44)	47.47–2522.24	− 0.510	0.610	NS
	Yes	681.92 (423.45–1056.56)	72.17–2940.2			
Runny nose	No	873.16 (235.92–1474.6)	47.47–2940.2	− 0.683	0.495	NS
	Yes	589.12 (462.4–1008.38)	72.17–2402.6			
Diarrhea	No	573.66 (334.36–1074.28)	47.47–2940.2	− 1.698	0.089	NS
	Yes	854.1 (604.76–1445.38)	265.32–2522.24			
Nausea	No	583.99 (342.06–1074.28)	63.74–2335.44	− 0.651	0.515	NS
	Yes	656.97 (334.36–1445.38)	47.47–2940.2			
Vomiting	No	583.99 (342.06–1074.28)	63.74–2335.44	− 0.697	0.486	NS
	Yes	656.97 (334.36–1445.38)	47.47–2940.2			
Abdominal pain	No	562.93 (335.28–1035.92)	72.17–2940.2	− 0.879	0.379	NS
	Yes	656.97 (462.4–1443.5)	47.47–2522.24			
Rash	No	623.7 (401.52–1074.28)	47.47–2940.2	− 0.315	0.753	NS
	Yes	604.76 (202.2–1445.38)	63.74–2335.44			
Conjunctivitis	No	599.54 (335.28–1038.84)	47.47–2940.2	− 1.100	0.271	NS
	Yes	1331.92 (342.06–2053.58)	75.83–2335.44			
Fatigue	No	537.82 (223.72–688.5)	63.74–1676.44	− 2.162	0.031	S
	Yes	786.12 (401.52–1443.5)	47.47–2940.2			
Headache	No	638.6 (335.28–1138.7)	47.47–2522.24	− 0.770	0.441	NS
	Yes	445.38 (342.06–675.34)	187.52–2940.2			
Muscle ache	No	589.12 (236.26–1074.28)	47.47–2522.24	− 0.964	0.335	NS
	Yes	733.02 (462.4–1218.46)	75.83–2940.2			
Joint pain	No	581.39 (334.36–1035.92)	47.47–2522.24	− 1.394	0.163	NS
	Yes	984.42 (445.38–1637.3)	75.83–2940.2			
Weight for age (Z-score)	Normal (-1 to + 2)	553.48 (236.26–1017.04)	47.47–2940.2	6.299‡‡	0.043	S
	Mildly underweight (-1 to -2)	944.32 (589.12–1676.44)	119.11– 2319.22			
	Moderately underweight (-2 to—3)	1022.15 (607.22–1812.5)	401.52–2335.44			

GDF-15:Growth differentiation factor 15

*IQR* Interquartile range’

^‡^Mann Whitney test

‡‡Kruskal Wallis test

P-value ≤ 0.05 = Significant (S), ˃0.05 is insignificant (NS)

**Table 3:** Statistical comparison according to parameters of the severity of studied children with COVID-19 as regards serum GDF-15 level

		Serum GDF-15 level (pg/ml)		Test value	P-value	Sig.
		Median (IQR)	Range			
PICU admission	No	453.89 (202.2–594.32)	47.47–2335.44	− 6.202	< 0.001	HS
	Yes	1296.02 (991.14–1871.58)	346.5–2940.2			
Duration of admission	Duration ≤ 10 days	521.26 (223.72–688.5)	47.47–2311.8	− 2.227‡	0.023	S
	Duration > 10 days	802.6 (401.52–1390.82)	119.11 –2940.2			
Comorbidities	No	521.26 (235.92–944.32)	47.47–2522.24	− 3.412‡	0.001	HS
	Yes	1035.92 (675.34–1443.5)	176.6–2940.2			
Clinical severity	Mild to Moderate	346.5 (202.2–537.82)	47.47– 854.1	49.047‡‡	< 0.001	HS
	Severe	999.76 (786.12–1218.46)	119.11–2335.44			
	Critically ill	1871.58 (1390.82–2319.22)	1038.84–2940.2			
Outcome	Non-survivors (N = 8)	1555.95 (1256.14–2420.73)	1038.84–2940.2	− 3.691‡	< 0.001	HS
	Survivors (N = 64)	570.59 (323.6–999.76)	47.47–2402.6			

GDF-15:Growth differentiation factor 15

*IQR* Interquartile range

^‡^Mann Whitney test; ‡‡: Kruskal Wallis test

P-value < 0.01: highly significant (HS); ≤ 0.05 is significant (S)

Serum GDF-15 had a significant positive correlation with ESR, LDH, D-dimer, PT, PTT, INR, AST, CK-MB, troponin I, and the number of lung lobes affected by CT chest. While it had a significant negative correlation with weight for age Z-score, oxygen saturation at room air, lymphocytic count, platelets count, and ejection fraction of the heart (Table 4).

**Table 4:** Statistical correlation between serum GDF-15 level with other studied parameters among studied patients with COVID-19

Studied parameters	Serum levels of GDF-15	
	Spearman’s correlation	P-value
Weight	− 0.047	0.693
Weight for age Z-Score	− 0.398^**^	**0.001**
Temperature	0.191	0.107
Heart rate	0.084	0.483
Oxygen saturation at room air	− 0.285^*^	**0.015**
Systolic blood pressure	− 0.023	0.849
Diastolic blood pressure	− 0.218	0.066
Respiratory Rate	0.137	0.252
TLC	− 0.128	0.286
Lymphocytes	− 0.303^**^	**0.010**
Neutrophils	0.006	0.963
Hemoglobin	− 0.157	0.189
Platelets	− 0.317^**^	**0.007**
ESR	0.369^**^	**0.001**
CRP	0.232*	**0.050**
LDH	0.238^*^	**0.044**
D-dimer	0.708^**^	**0.000**
Ferritin	0.228	0.054
PT	0.487^**^	**0.000**
PTT	0.369^**^	**0.001**
INR	0.365^**^	**0.002**
ALT	0.077	0.522
AST	0.345^**^	**0.003**
BUN	− 0.006	0.963
Creatinine	0.088	0.463
CK-Total	0.226	0.056
CK-MB	0.570^**^	**0.000**
Troponin I	0.639^**^	**0.000**
Number of lobes affected in chest CT	0.425^**^	**0.000**
Ejection Fraction (Echo)	− 0.593^**^	**0.000**

*GDF-15*Growth differentiation factor 15, *TLC* Total Leucocytic Count; *ESR* Erythrocyte sedimentation rate, *CRP* C-reactive protein, *LDH* lactate dehydrogenase, *PT* Prothrombin Time; *PTT* Partial Thromboplastin Time, *INR* International normalized ratio, *ALT* Alanine Aminotransferase; *AST* Aspartate aminotransferase, *BUN* Blood Urea Nitrogen; *CK* Creatine kinase; *CK-MB* Creatine kinase MB, *CT* computerized tomography, *ECHO* echocardiography

P-value < 0.01: highly significant; ≤ 0.05 is significant; ˃0.05 is insignificant

ECHO findings show that there was valvular affection in 33.3% of patients which was mainly tricuspid regurge (20.8%). Cardiomegaly was found in 26.4% of patients presented mainly as left ventricular dilatation and hypertrophy (23.6%). Other less common findings were: pulmonary hypertension in 5.6%, and pericardial effusion in 4.2%. Also, there was a highly statistically significant difference in serum GDF-15 level among patients who had cardiac affection including; Echo abnormalities, presence of carditis, and low ejection fraction, compared to those who hadn’t (Table 5).

**Table 5:** Statistical comparison according to ECHO findings and cardiac affection of studied children with COVID-19 as regards serum GDF-15 level

		Serum GDF-15 level (pg/ml)		Test value	P-value	Sig.
		Median (IQR)	Range			
ECHO	Abnormal	1008.38 (530.27–1445.38)	75.83–2940.2	− 4.089‡	< 0.001	HS
	Normal	342.06 (202.2–604.76)	47.47–1637.3			
Carditis	No	558.34 (265.32–873.16)	47.47–2940.2	− 3.814‡	< 0.001	HS
	Yes	1443.5 (1138.7–1676.44)	346.5–2335.44			
Ejection Fraction	Low	1443.5 (1038.84–2150.4)	119.11–2940.2	− 5.706‡	< 0.001	HS
	Normal	521.26 (236.26–638.6)	47.47–2335.44			
Cardiac affection	No	502.53 (235.92–604.76)	47.47–1017.04	− 6.425‡	< 0.001	HS
	Yes	1444.44 (1074.28–2150.4)	119.11–2940.2			

GDF-15:Growth differentiation factor 15,

IQR: Interquartile range

^‡^Mann Whitney test

P-value < 0.01: highly significant (HS)

Regarding the validity of serum GDF-15 level to discriminate children with COVID-19 from controls, its sensitivity was 79.17%, and its specificity was 100.0%, at a cut-off > 272.8 pg/mL. While, regarding the validity of serum GDF-15 level to assess cardiac affection of COVID-19 children, its sensitivity was 92.31%, and its specificity was 95.74% at a cut-off > 873.16 pg/mL. Regarding the validity of serum GDF-15 level to discriminate patients with different clinical severity grading (mild to moderate from severe and critically ill), its sensitivity was 88.57%, and its specificity was 97.3% at cut-off > 688.5 pg/mL (Table 6). Using multivariate linear regression analysis, higher GDF-15 was associated with lower ejection fraction and higher INR in COVID-19 children (Table 7).

**Table 6:** Validity (AUC, sensitivity, specificity) for serum GDF-15 level to detect COVID-19 in pediatric patients, detect cardiac affection, and detect clinical severity

Parameter	AUC	P-value	Cut of Point (pg/ml)	Sensitivity	Specificity	PPV	NPV
Serum GDF-15 level to detect COVID-19	0.910	< 0.001	> 272.8	79.17	100.0	100.0	82.8
Serum GDF-15 level to detect cardiac affection	0.958	< 0.001	> 873.16	92.31	95.74	92.3	95.7
Serum GDF-15 level to detect clinical severity	0.957	< 0.001	> 688.5	88.57	97.30	96.9	90.0

AUC Area under a Curve, (P- value**)** Probability value, (NPV**)** Negative predictive value, (PPV**)** Positive predictive value. GDF-15:Growth differentiation factor 15

P-value < 0.01: highly significant (HS)

**Table 7:** Multivariate linear regression analysis for predictors of serum GDF-15 level

	Unstandardized Coefficients		Standardized Coefficients	t	P-value
	B	SE	Beta		
Weight for age Z-Score	− 35.024	58.018	− 0.060	− 0.604	0.548
Oxygen saturation (Room air)	− 4.141	19.205	− 0.021	− 0.216	0.830
Lymphocytes	− 3.638	19.975	− 0.017	− 0.182	0.856
Platelets	− 0.104	0.465	− 0.021	− 0.224	0.824
ESR	− 1.781	2.303	− 0.086	− 0.774	0.442
CRP	0.551	0.970	0.068	0.568	0.572
LDH	− 0.201	0.294	− 0.081	− 0.683	0.498
D-dimer	11.199	21.072	0.066	0.531	0.597
INR	431.613	149.510	0.273	2.887	0.005
AST	0.713	0.442	0.150	1.614	0.112
CK-MB	− 0.872	1.608	− 0.051	− 0.542	0.590
Troponin I	185.353	329.763	0.065	0.562	0.576
Number of lobes Affected	86.402	48.551	0.195	1.780	0.080
Ejection Fraction	− 31.598	9.301	− 0.465	− 3.397	0.001

*ESR* Erythrocyte sedimentation rate, *CRP* C-reactive protein, *INR* The international normalized ratio, *AST* aspartate aminotransferase, *CK-MB* Creatine kinase MB

## Discussion

GDF-15 elevated alongside other inflammatory biomarkers in COVID-19 patients. Compared to other inflammatory markers including CRP, ferritin, and D-dimer, GDF-15 was more sensitive and specific in diagnosing the early stage of COVID-19 severity and admission to the ICU in seriously afflicted COVID-19 patients. Myhre et al. (2020) study on 123 adult patients found that the GDF-15 serum level was extremely sensitive, specific, and superior to other known inflammatory and cardiovascular biomarkers, and it is linked to ICU admission of patients with severe disease [15].

Fatigue and dyspnea are common symptoms related to COVID-19 and Long COVID that could last 12 weeks postinfection [16]. In our study, Serum GDF-15 was significantly higher in children with dyspnea (P = 0.043), and fatigue (P = 0.031). Myhre et al. [15] also found a positive correlation between serum GDF-15 levels and the presence of fatigue in COVID-19 patients, which strongly supports the role of GDF-15 as a biomarker of muscular and myocardial affection in COVID-19 patients and could suggest the potential role of GDF-15 as a therapeutic target for Long COVID fatigue.

The study of Alserawan et al. [11] on adult patients with COVID-19 found a significant correlation between GDF-15 and other inflammatory biomarkers of COVID-19 severity as CRP, D-dimer, and lymphopenia, which were similar to our results in the pediatric population. Also, in our study, ESR was elevated in 65.3% of patients and this correlated positively with the serum level of GDF-15. This agreed with other studies such as Tanrikulu et al. [17] and Wischhusen et al. [10] both found a positive correlation between GDF-15 and ESR.

Although increased ferritin is a poor predictor of COVID-19 outcome [5], we could not prove its correlation with the GDF-15 level in our study. This is in contrast with the studies of Luis et al. [18], and Alserawan et al. [11] which found a significant positive correlation between GDF-15 levels and ferritin (r = 0.334, 0.285; p = 0.006, 0.013, respectively).

LDH is an enzyme found in nearly all living cells and plays a role in energy production. It is released into the bloodstream when there is cell damage or destruction. Elevated LDH levels can be seen in various diseases and conditions, including heart disease, liver disease, muscle injury, and hemolysis [19]. GDF-15 and LDH are not typically directly correlated, but their levels can be influenced by similar underlying conditions or disease processes.

Coagulation abnormalities, including elevated D-dimer and prolonged PT, INR, and PTT, have been observed in some severe cases of COVID-19, both in children and adults. These abnormalities are indicative of a hypercoagulable state and an increased risk of blood clot formation. Rochette et al. [3] suggested the role of GDF-15 in causing microvascular injury and endothelitis that could explain its role in cardiovascular injury.

In COVID-19, severe cases can lead to acute respiratory distress syndrome (ARDS) and hypoxia, where the body's oxygen levels are significantly reduced. Hypoxia is a serious complication of COVID-19 and can lead to significant tissue damage and organ dysfunction. GDF-15 is predominantly located in endothelial cells and is upregulated by hypoxia. GDF-15 expression is induced by lung injury and is suggested to be a hallmark of tissue injury in many organs [20]. This study showed that serum levels of GDF-15 were significantly correlated with oxygen saturation in room air. There was a positive correlation between the GDF-15 level and the degree of hypoxemia, in addition to a significant increase in the GDF-15 level of patients who presented with dyspnea and those with more affected lung lobes in chest CT. This shows that GDF-15 may be able to detect silent hypoxemic patients, a COVID-19 sign that has been linked to significantly higher risk [15].

The present study showed that GDF-15 levels correlated positively with both CK-MB and troponin I levels (r = 0.570 and 0.639 respectively), and this agreed with the study of Wallentin et al. [21] and Eindhoven et al. [22]. Also, we found a positive correlation between GDF-15 level and cardiac affection. This agreed with Eggers et al. [23] who found that GDF-15 exhibited the strongest incremental value to cardiovascular risk indicators, both in terms of prognostic discrimination and reclassification of cardiovascular risks (95% CI, 0.71 (0.67–0.76)) in comparison to NT-proBNP (P value < 0.001 and 0.040, respectively) in COVID-19 pathophysiology [24]. Similarly, Myhre et al. [15] found that GDF-15 correlated positively with cardiovascular biomarkers, cardiac Troponin (cTnT), and pro-B- type natriuretic peptide (pro-BNP); with correlation coefficients (0.52 and 0.49 respectively) and p < 0.05 for both.

The study of Hacıoğlu et al. [25] found that GDF-15 was detected to be significantly increased in patients with subtle changes in diastolic blood pressure (DBP), end-diastolic volume (EDV), stroke volume (SV), and cardiomegaly. Similarly, Roenningen et al. [26] found that GDF-15 was valuable in detecting the incidence of atrial fibrillation in patients with left atrial structural remodeling and hypertrophy. In addition, Lockhart et al. [27] found that the production of GDF-15 is upregulated in cardiomyocytes following ischemia–reperfusion injury. Also, this high expression has been reported after MI leading to the suggestion that GDF-15 may be a biomarker for heart failure.

In our study, plasma GDF-15 levels were significantly higher in non-survivors in comparison to survivors indicating that it’s a good predictor of mortality. Similarly, the study of Luis et al. [18], Myhre et al. [15], Ahmed et al. [28], and Teng et al. [29] found that the GDF-15 level was significantly higher in COVID-19 patients who died, suggesting its potential role in evaluating the prognosis of patients. Dynamic changes in GDF-15 levels reflected disease progression, with high levels linked to symptom deterioration, followed by a dramatic decline in plasma GDF-15 levels at the time of clinical and radiological improvement and discharge. This study indicates that GDF-15 could be used as a predictor of the progression of the disease [30]. Our cut-off value to detect severity was > 688.5 which is much lower than that detected by Myhre et al. [15] in adult patients with COVID-19 (2252 pg/ml). Also, Hongisto et al. [31] found that ˃7000pg/ml is the cutoff of GDF-15 to predict mortality in adults with cardiogenic shock. Our cutoff level to detect cardiac affection (> 873.16 pg/ml) is also lower than Tantawy et al. [32] which suggested 1500 pg/ml for differentiating patients with heart disease from those without (the study included children and adults without COVID-19). So, more studies are needed to predict the prognostic cutoff value of GDF-15 in pediatrics and adults.

The limitations of our study: 11 cases (15.38%) had comorbid diseases such as renal disease, cerebrovascular disease, and diabetes, and these cases had higher GDF-15 than those with COVID-19 without comorbidities which can probably indicate more severe disease but the effect of the comorbid disease on GDF-15 could not be excluded. Also, our study included only hospitalized children with COVID-19 without including non-hospitalized children.

Based on these findings, GDF-15, which is easily available by commercial assays on large automated analytic platforms, may represent a clinically useful risk stratification tool that provides important pathophysiological insights in patients hospitalized with COVID-19. Thus, in real-life situations with hospitalized COVID-19 patients, serial measurements of GDF-15 can be an aid in patient triage and follow disease progression more efficiently.

## Conclusion

GDF15 is emerging as a promising field for research in the context of children with COVID-19. Its role as a potential biomarker for disease severity and a therapeutic target should be explored to improve our understanding and management of COVID-19 in the pediatric population. Further research and clinical trials are necessary to determine the utility and safety of GDF15-related interventions in children with COVID-19.

## Data Availability

Data are available upon request, by keeping the confidentiality of our patient.

## Abbreviations

ACE2: Angiotensin-converting enzyme 2
AUC: The area under the curve
CK-MB: Creatinine kinase-MB
CO-RADS: COVID-19 Reporting and Data System
COVID 19: Cornavirus disease 19
CRP: C- reactive protein
cTnT: Cardiac troponin
DBP: Diastolic blood pressure
EDV: End-diastolic volume
ESR: Erythrocyte sedimentation rate
GDF-15: Growth differentiation factor-15
ICU: Intensive Care Unit
IL-6: Interleukin-6
IQR: Interquartile range
MI: Myocardial infarction
NPV: Negative predictive value
PPV: Positive predictive value
pro-BNP: Pro-B-type natriuretic peptide
P value: Probability value
RT-PCR: Real time-PCR
SD: Standard deviation
SV: Stroke volume
TGFβ: Transforming Growth Factor β

## References

[1] Kang Y, Chen T, Mui D, Ferrari V, Jagasia D, Scherrer-Crosbie M (2020). Cardiovascular manifestations and treatment considerations in COVID-19. Heart.

[2] Zumla A, Hui DS, Azhar EI, Memish ZA (2020). Maeurer M reducing mortality from 2019-nCoV: host-directed therapies should be an option. Lancet.

[3] Rochette L, Dogon G, Rigal E, Zeller M, Vergely C, Cottin Y (2022). GDF15: a modulator of immunity and a predictive biomarker of cardiovascular events: a strategy in COVID-19. Ann Cardiol Angeiol.

[4] Atlas HP. 2021: https://www.proteinatlas.org/. ENSG00000164904-ALDH7A1/tissue Accessed June 1 2023.

[5] Chang JY, Hong HJ, Kang SG, Kim JT, Zhang BY, Shong M (2020). The Role of growth differentiation factor 15 in energy metabolism. Diabetes Metab J.

[6] Ost M, Igual Gil C, Coleman V, Keipert S, Efstathiou S, Vidic V (2020). Muscle-derived GDF15 drives diurnal anorexia and systemic metabolic remodeling during mitochondrial stress. EMBO Rep.

[7] Johann K, Kleinert M, Klaus S (2021). The role of GDF15 as myomitokine. Cells.

[8] Jojo R, George S (2023). Yap; Emerging roles of growth differentiation factor 15 in immunoregulation and pathogenesis. J Immunol.

[9] Barbalho SM, Minniti G, Miola VFB, Haber JFdS, Bueno PCdS, de Argollo Haber LS, Girio RSJ, Detregiachi CRP, Dall’Antonia CT, Rodrigues VD (2023). Organokines in COVID-19: a systematic review. Cells.

[10] Wischhusen J, Melero I, Fridman WH (2020). Growth/differentiation factor-15 (GDF-15): from biomarker to novel targetable immune checkpoint. Front Immunol.

[11] Alserawan L, Peñacoba P, Orozco Echevarría SE, Castillo D, Ortiz E, Martínez-Martínez L (2021). Growth differentiation factor 15 (GDF-15): a novel biomarker associated with poorer respiratory function in COVID-19. Diagnostics.

[12] Alp EE, Dalgic N, Yilmaz V, Altuntas Y, Ozdemir HM (2021). Evaluation of patients with suspicion of COVID-19 in pediatric emergency department. Sisli Etfal Hastan Tip Bul.

[13] Ayatollahi SMT (1995). Age standardization of weight-for-height in children using a unified Z-score method. Ann Hum Biol.

[14] Bayramoglu Z, Canıpek E, Comert RG, Gasimli N, Kaba O, Sarı Yanartaş M (2021). Imaging features of pediatric COVID-19 on chest radiography and chest CT: a retrospective. Single-Center Study Acad Radiol.

[15] Myhre PL, Prebensen C, Strand H, Røysland R, Jonassen CM, Rangberg A (2021). Growth differentiation factor 15 provides prognostic information superior to established cardiovascular and inflammatory biomarkers in unselected patients hospitalized with COVID-19. Circulation.

[16] Gross M, Lansang NM, Gopaul U, Ogawa EF, Heyn PC, Santos FH, Sood P, Zanwar PP, Schwertfeger J, Faieta J (2023). What do i need to know about long-covid-related fatigue, brain fog, and mental health changes?. Arch Phys Med Rehabil.

[17] Tanrikulu O, Sariyildiz MA, Batmaz I, Yazmalar L, Polat N, Kaplan I (2017). Serum GDF-15 level in rheumatoid arthritis: relationship with disease activity and subclinical atherosclerosis. Acta Reumatol Port.

[18] de Luis García GR, Mulero MDR, Olivo MH, Rojas CR, Arenas VR, Morales MG (2012). Circulating levels of GDF-15 and calprotectin for prediction of in-hospital mortality in COVID-19 patients: a case series. J Infect..

[19] Farhana A, Lappin SL. Biochemistry, Lactate Dehydrogenase. [Updated 2023 May 1]. In: StatPearls. Treasure Island (FL): StatPearls Publishing; 2023 Jan-. https://www.ncbi.nlm.nih.gov/books/NBK557536/32491468

[20] Zimmers TA, Jin X, Hsiao EC, McGrath SA, Esquela AF, Koniaris LG (2005). Growth differentiation factor-15/macrophage inhibitory cytokine-1 induction after kidney and lung injury. Shock.

[21] Wallentin L, Lindbäck J, Eriksson N, Hijazi Z, Eikelboom JW, Ezekowitz MD (2020). Angiotensin-converting enzyme 2 (ACE2) levels in relation to risk factors for COVID-19 in two large cohorts of patients with atrial fibrillation. Eur Heart J.

[22] Eindhoven JA, Van den Bosch AE, Oemrawsingh RM, Baggen VJ, Kardys I, Cuypers JA (2016). Release of growth-differentiation factor 15 and associations with cardiac function in adult patients with congenital heart disease. Int J Cardiol.

[23] Eggers KM, Kempf T, Larsson A, Lindahl B, Venge P, Wallentin L (2016). Evaluation of temporal changes in cardiovascular biomarker concentrations improves risk prediction in an elderly population from the community. Clin Chem.

[24] Wollert KC, Kempf T, Wallentin L (2017). Growth differentiation factor 15 as a biomarker in cardiovascular disease. Clin Chem.

[25] Hacıoğlu, Yalçın & Pişkinpaşa, Mehmet & Kılıçkaya, Pelin & Niyazoğlu, Mutlu & Hacıoğlu, Burcu & Hatipoglu, et al. 2022. Increased Serum Growth Differentiation Factor 15 Levels may be Associated with Diastolic Dysfunction in Acromegaly. Istanbul Med J. 23. 179–182. 10.4274/imj.galenos.2022.44788.

[26] Rønningen PS, Solberg MG, Berge T, Brynildsen J, Aagaard EN, Kvisvik B (2022). Prediction of incident atrial fibrillation with cardiac biomarkers and left atrial volumes. Heart.

[27] Lockhart SM, O’Rahilly S (2019). The wasting hormone GDF15 frees up fat to fight infection. Nat Metab.

[28] Ahmed DS, Isnard S, Berini C, Lin J, Routy JP, Royston L (2022). Coping with stress: the mitokine GDF-15 as a biomarker of COVID-19 severity. Front Immunol.

[29] Teng X, Zhang J, Shi Y, Liu Y, Yang Y, He J (2021). Comprehensive profiling of inflammatory factors revealed that growth differentiation factor-15 is an indicator of disease severity in COVID-19 patients. Front Immunol.

[30] Gupta A, Madhavan MV, Sehgal K, Nair N, Mahajan S, Sehrawat TS (2020). Extrapulmonary manifestations of COVID-19. Nat Med.

[31] Hongisto M, Kataja A, Tarvasmäki T, Holopainen A, Javanainen T, Jurkko R, Jäntti T, Kimmoun A, Levy B, Mebazaa A, Pulkki K, Sionis A, Tolppanen H, Wollert KC, Harjola VP, Lassus J, CardShock investigators (2019). Levels of growth differentiation factor 15 and early mortality risk stratification in cardiogenic shock. J Card Fail.

[32] Tantawy AA, Adly AA, Ismail EA, Youssef OI, Ali ME (2015). Growth differentiation factor-15 in children and adolescents with thalassemia intermedia: relation to subclinical atherosclerosis and pulmonary vasculopathy. Blood Cells Mol Dis.

